# Case Report: Notoedric Mange and Aelurostrongylidosis in Two Domestic Cats From Rural Environment in Romania

**DOI:** 10.3389/fvets.2022.849525

**Published:** 2022-06-01

**Authors:** Adriana Györke, Mirabela Oana Dumitrache, Aurora Livia Ursache, Gianluca D'Amico, Viorica Mircean

**Affiliations:** ^1^Parasitology and Parasitic Diseases Department, Faculty of Veterinary Medicine, University of Agricultural Sciences and Veterinary Medicine, Cluj-Napoca, Romania; ^2^Dermatology Clinic, Faculty of Veterinary Medicine, University of Agricultural Sciences and Veterinary Medicine, Cluj-Napoca, Romania

**Keywords:** cat, mange, lungworm, *Notoedres*, *Aelurostrongylus*

## Abstract

This article describes two cases of notoedric mange concurrent with aelurostrongylidosis in two domestic-owned cats from a rural environment in Romania. Two European shorthair cats originating from the same litter were referred to our clinic, at 2 months apart, with a history of skin lesions, pruritus, weight loss, and respiratory signs. Cats lived mainly outdoor together with the queen and a third littermate. The latter two expressed the same clinical signs and had died before the presentation of the first cat. None of the four cats was vaccinated, dewormed, or treated for external parasites. Coat brushing, skin scrapings, skin cytology, earwax direct microscopic examination, and coproparasitological techniques (flotation and Baermann methods) were used as laboratory procedures. A blood sample was also collected for hematology, blood biochemistry, and feline immunodeficiency virus/feline leukemia virus (FIV/FeLV) test. *Notoedres cati* mites in high numbers were identified by all complementary tests, alongside fleas and *Aelurostrongylus abstrusus* first-stage larvae. The blood analysis revealed neutrophilia, and the FIV/FeLV fast test was negative. The cats were successfully treated off-label with selamectin spot-on formulation (Stronghold^®^, Zoetis) three times at 1- or 4-week intervals. Furthermore, they were treated with amoxicillin trihydrate/clavulanic acid, housed indoor, and fed with a commercial diet. Before presentation to the clinic, the female cat was unsuccessfully treated with a combination of fipronil, S-methoprene, eprinomectin, and praziquantel. During this period, the female cat remained outdoor and fed with home wastes. The cats become negative for *A. abstrusus* L1 larvae after 2–4 months of treatment. The owners developed pruritic skin lesions 1 month after introducing the first cat in the house. In conclusion, notoedric mange and aelurostrongylidosis can be treated successfully with selamectin as a spot-on formulation and the treatment must be continued until no parasite will be detected through specific techniques. The success of treatment depends on improving the quality of animal life (nutrition and hygiene) and treatment of secondary complications.

## Introduction

Notoedric mange is a pruritic and contagious skin disease caused by the burrowing mites belonging to the *Notoedres* genus (family Sarcoptidae). Nowadays, more than forty-one species are recognized in the *Notoedres* genus (1). *Notoedres* mites affect mammals belonging to Rodentia, Carnivora, Chiroptera, and Lagomorpha orders (2). *Notoedres cati* is the first species from the genus described by Hering in 1838 (2). *Notoedres cati* affects mainly cats, but it was found in more than 18 host mammal species (insectivores, rodents, lagomorphs, bobcats, procyonids, and viverrids), including humans (3). It is transmitted by direct contact and rarely indirectly from the contaminated environment (4). All stages (eggs, larvae, nymphs, and adults) of the mite live in the skin and do not survive out of the host (3, 4). The life cycle of *N. cati* is similar to *Sarcoptes scabiei*, but a detailed description is not available (5). The larvae, nymphs, and adults of *N. cati* borrow tunnels in the superficial epidermis, on the face and ears, and sometimes on the legs and genital regions (3, 6). After mating on the skin surface, female mites burrow tunnels within the horny layer of the skin, where they lay 2–3 eggs a day. The life cycle lasts 14–21 days, in favorable environmental conditions.

Notoedric mange is considered a rare skin condition in cats and is mainly diagnosed in stray cats (4). Little information is available on this topic, represented mainly by case reports and therapeutical trials, and few epidemiological studies. In Romania, the parasite was previously reported in 3 out of 24, and in 1 out of 389 cats with skin lesions (7, 8). More recently, a case report of notoedric mange in association with otodectic mange and roundworm infection was reported (9).

Cats with notoedric mange present intense pruritus and skin lesions such as papules, alopecia, erythema, excoriations, scales, crusts, and lichenification (4, 6). The first lesions appear on the edge of the ear pinna and they spread fast on the head and neck, and sometimes on the legs and perineum facilitated by the cat's habits of self-grooming and of sleeping in a curled position (10). Untreated diseases can be fatal both in young and adult cats (4). Notoedric mange is successfully treated with macrocyclic lactones (11).

Aelurostrongylidosis is a common verminous pneumonia caused by metastrongyloid lungworm *Aelurostrongylus abstrusus* in cats worldwide (12). The clinical presentation varies from subclinical to severe diseases, but most of the animals present mild to moderate respiratory signs such as sneezing and nasal discharge, cough, dyspnea, tachypnea, and abdominal breathing (13). Macrocyclic lactones and fenbendazole are commonly used to treat aelurostrongylidosis (11, 13).

This report describes two cases of notoedric mange concurrent with aelurostrongylidosis in domestic-owned cats from a rural environment in Romania.

## Case History

Two European shorthair cats originating from the same litter were referred to the Faculty of Veterinary Medicine Cluj-Napoca, at the Dermatology Clinic, at 2 months apart, with a history of skin lesions, weight loss, and respiratory signs (sneezing and cough). Both cats lived in a rural area (Alba County), mainly outdoor together with the queen and a third littermate. The latter two died a month before the consultation of the first cat in our clinic, and they had expressed the same clinical signs. None of the four cats was vaccinated, dewormed, or treated for external parasites. Each case will be presented below.

### Case 1

**Figure 1:**
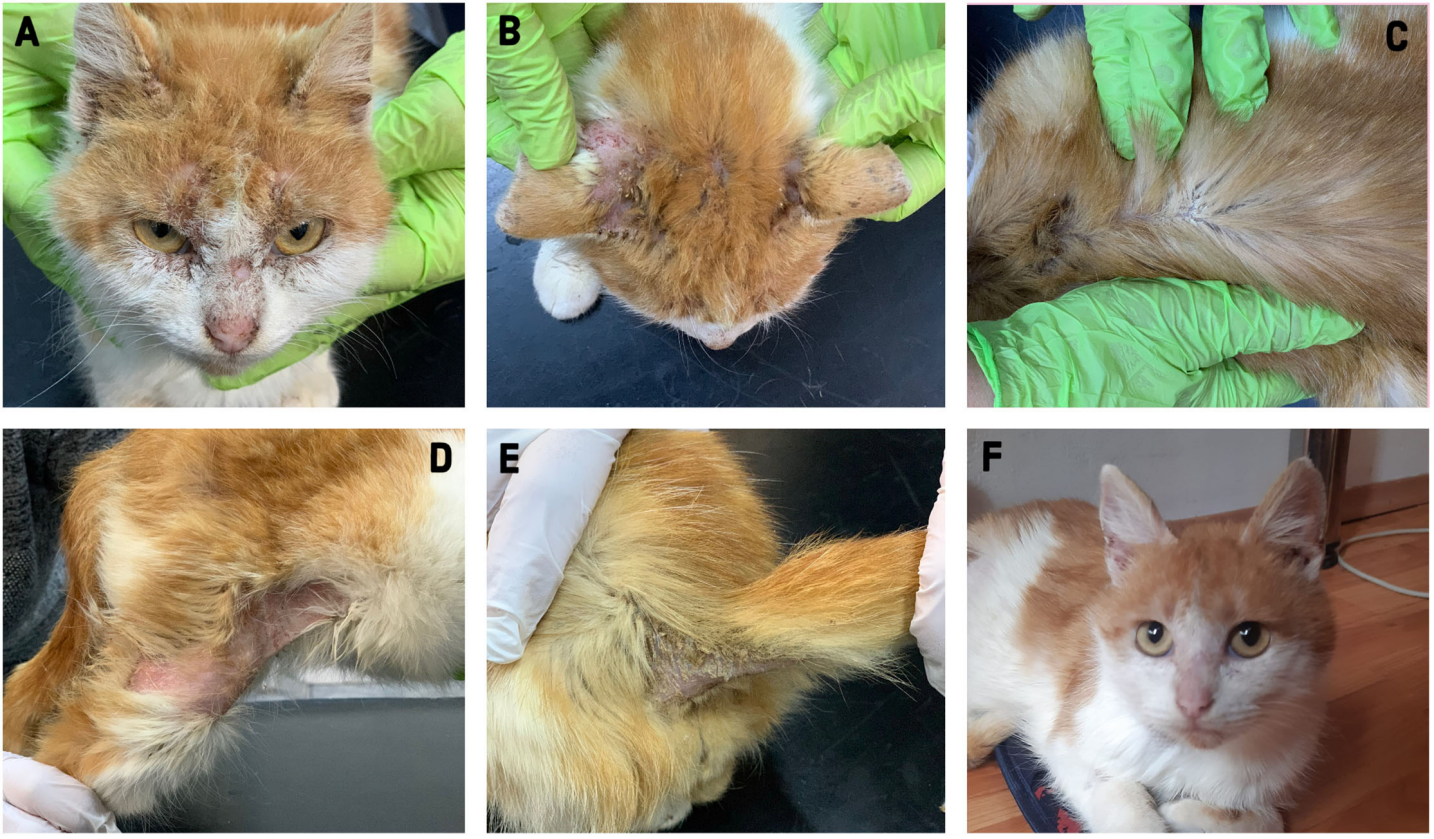
Clinical aspect of the 18-month-old male cat (case 1) before **(A–E)** and after **(F)** treatment.

In January 2021, an 18-months-old male cat was submitted to the clinic. The physical examination revealed poor body condition (body score 3/9, 1.5 kg), a body temperature of 37.0°C, skin lesions, excessive earwax, moderate head pruritus, fleas, and flea feces. Alopecia, erythema, scales, crusts, and lichenification were found on the head (face and ears pinna), dorsal thorax, right hind limb, tail, and ventral part of the tail (Figure 1). Based on the history and clinical examination, the main differential diagnoses were flea allergy dermatitis and ectoparasitic infestation such as lice (*Felicola subrostrata*) and/or mites (*Otodectes cynotis, N. cati, Cheiletiella* spp.). Coat brushing, skin scrapings, skin cytology (scotch test), earwax direct microscopic examination, and coproparasitological techniques (flotation and Baermann methods) were used as laboratory procedures. A blood sample was also collected for hematology, blood biochemistry, and feline immunodeficiency virus/feline leukemia virus (FIV/FeLV) test (SNAP FIV/FeLV Combo Test, IDEXX).

**Figure 2:**
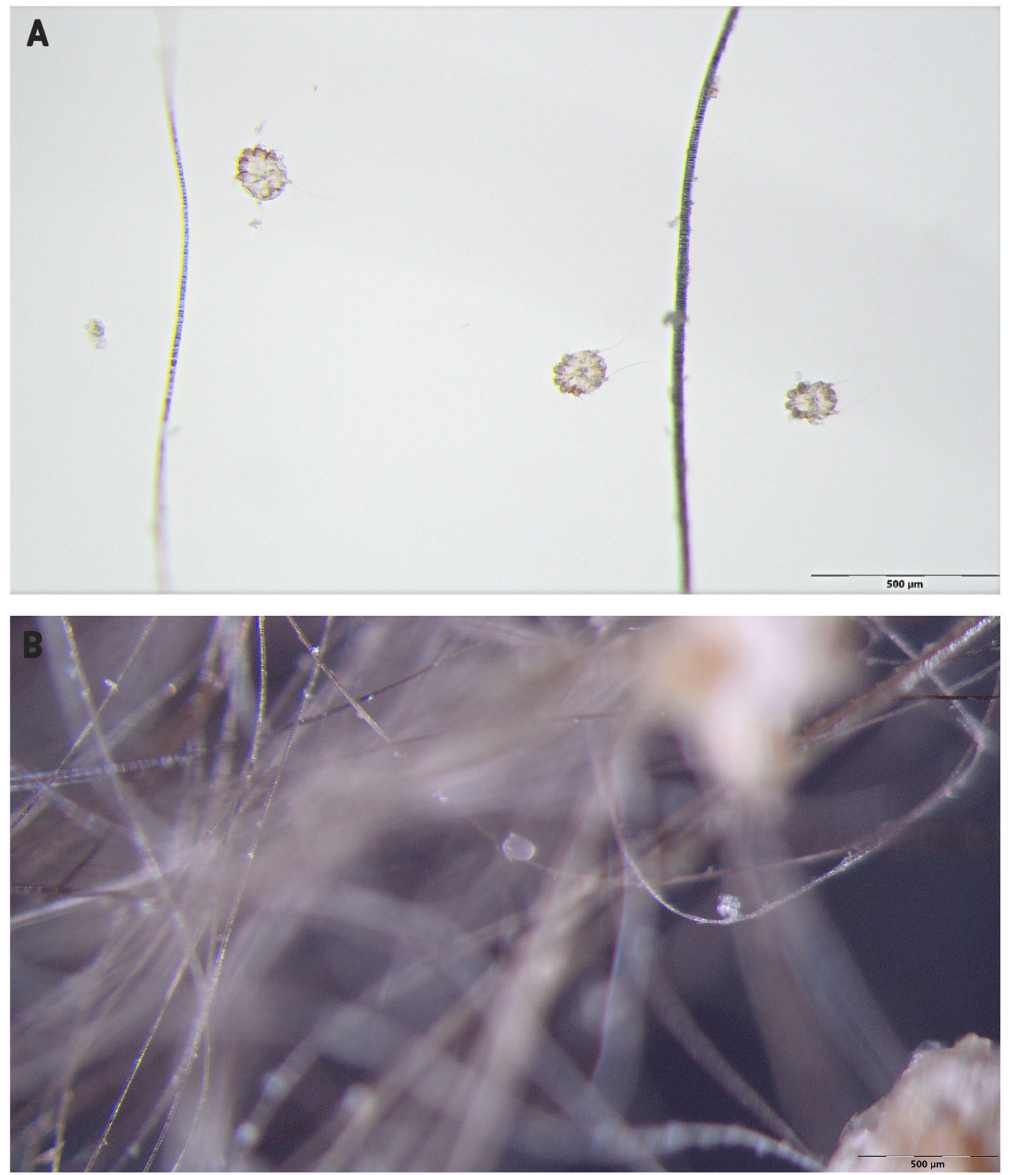
*Notoedres cati* identified through an stereomicroscope examination of the coat brushing (**A**, adults; **B**, egg).

**Figure 3:**
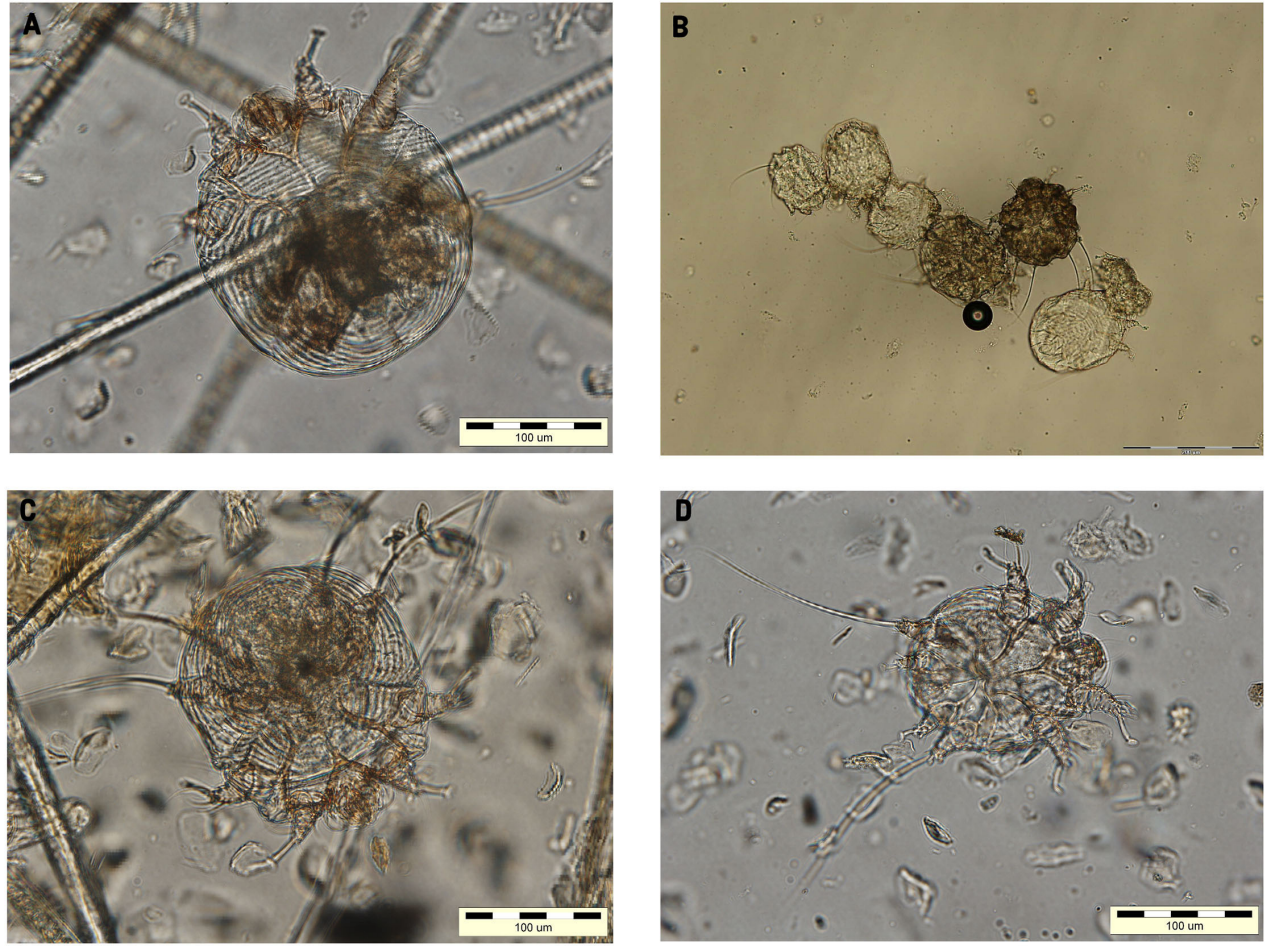
*Notoedres cati* (x100) identified through skin scraping **(A,C,D)** and flotation **(B)**.

A stereomicroscope examination of the coat brushing revealed the presence of fleas (adults and flea feces), adult mites, and eggs of *Notoedres cati* in high numbers (Figure 2). *N. cati* mites were morphologically identified based on the presence of concentric rings on the dorsal idiosoma and subterminal anus (Figure 3) (4). Furthermore, eggs and adult mites of *N. cati* were detected by the skin scrapings, cytology, and coproparasitological examination by flotation technique (Figure 3). The microscopic examination of the earwax was negative. *Aelurostrongylus abstrusus* first stage larvae (L1) and *N. cati* mites were identified by the Baermann technique. The presence of *A. abstrusus* L1 was confirmed by multiplex PCR (mPCR) of internally transcribed spacer 2 (ITS2) region on recovered larvae by Baerman technique following the protocol previously described (14). Coci were identified to the cytology. A blood analysis revealed neutrophilia. The FIV/FeLV fast test was negative.

After diagnosis, the cat was housed indoor in a different location (Cluj County), and the current diet (leftover food) was changed with a commercial dry food. The cat was off-label treated with selamectin (Stronghold^®^, Zoetis) three administration (Supplementary File 1), amoxicillin trihydrate/clavulanic acid (Kesium^®^, Ceva) for 10 days, and single administration of praziquantel and pyrantel embonate (Cestal Cat^®^, Ceva). The skin scrapings and coproparasitological examination by flotation and Baermann technique were repeated 2 weeks after the first treatment and 4 weeks after the second and third treatments, respectively (Supplementary File 1).

Clinical improvement was noticed at 2 weeks after the first treatment with selamectin; the cat gained weight (body score 4/9), the pruritus decreased, and alopecia and scales was noticed on the head and right hind limb. *Notoedres cati* mites were detected by the skin scrapings and flotation in low numbers. The Baermann technique revealed an increased number of *A. abstrusus* larvae compared with the first examination (Supplementary File 1). After a month from the second treatment, skin lesions were observed only on the ear pinna (diffuse alopecia, hyperpigmented macules, and scales) and no pruritus was present. A single mite was detected by skin scraping. Although in a low number, larvae of *A. abstrusus* were still detected by the Baermann technique. After 4 weeks from the third treatment, the cat had no clinical signs or lesions (Figure 1). Both the skin scrapings and the Baermann technique were negative (Supplementary File 1). One month after the cat was diagnosed with notoedric mange, the onset of pruritic skin lesions (papules) on the chest were reported by the owners.

### Case 2

Due to the contagious character of the notoedric mange, and considering the epidemiological characteristics of *A. abstrusus* as well as the similar clinical manifestations, it was presumed that the surviving littermate was suffering from the same conditions. A combination of labeled fipronil, S-methoprene, eprinomectin, and praziquantel (Broadline^®^, Boehringer Ingelheim) two times monthly was administrated at our recommendation.

**Figure 4:**
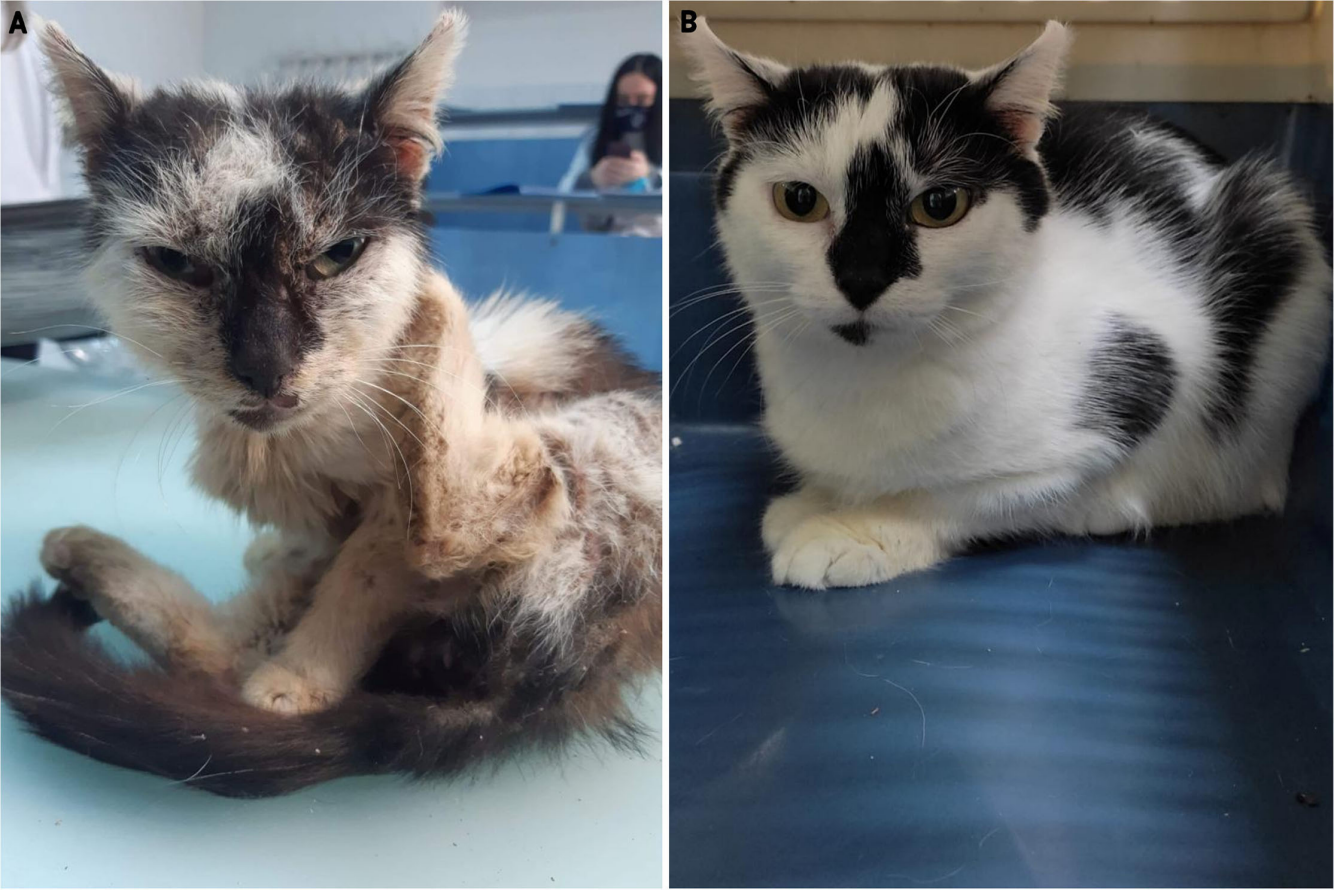
Clinical aspect of the 20-month-old female cat (case 2) before **(A)** and after **(B)** treatment.

In March 2021, the surviving littermate, a 20-month-old female was referred to our clinic because no clinical improvement was obtained after the treatment with fipronil, S-methoprene, eprinomectin, and praziquantel (Broadline^®^, Boehringer Ingelheim). The clinical examination of the second cat revealed poor body condition (3/9, weight 900 g), cough, sneeze, pruritus, and skin lesions such as diffuse alopecia, scales, crusts, erosions, and lichenification (Figure 4). The skin lesions were distributed on the head (face, ear pinna), lateral neck, lateral thorax, ventrum, hind limbs, lumbosacral area, and ventral part of the tail. The same diagnostic techniques as for the first cat were used. Large numbers of *N. cati* and *A. abstrusus* L1 larvae were identified. After the first consultation, the cat was housed indoors, the diet was changed with a commercial food, and she was treated with selamectin (Stronghold^®^, Zoetis) every 2 weeks, for 6 weeks, and then monthly until negative results to the Baermann technique (Supplementary File 2). An antibiotic (amoxicillin trihydrate/clavulanic acid; Kesium^®^, Ceva) was prescribed for 10 days. Clinical improvement was registered after 2 weeks from the first treatment and a complete clinical cure in a month (Figure 4). Skin scrapings became negative after 4 weeks (Supplementary File 2). The cat became negative for *A. abstrusus* in 4 months (Supplementary File 2).

## Discussion

The most common primary pruritic skin conditions in cats are represented by ectoparasites and allergies that are frequently complicated by bacterial or fungal infections (15). Our littermate cats presented pruritic skin lesions and respiratory signs. They had outdoor access, lived in a rural area, and they did not benefit from any vaccination and parasite prevention treatment.

*Notoedres cati* affects both the health and wellbeing of cats. The pruritic skin lesions can rapidly aggravate as a consequence of scratching (up to self-mutilation) and secondary bacterial infections. If it is left untreated in kittens and immunosuppressed individuals, notoedric mange can cause death in 4–5 months (3, 4, 10). Clinically, cases are rare and are diagnosed mainly in cats kept or living in poor hygienic conditions, with malnutrition, and with concurrent diseases (4). In Europe, the prevalence of the diseases varies between 0.2% in owned cats and 2.4% in stray ones (16–18).

The cats from this report presented a generalized clinical form and most probably got the infection from their queen who died together with a littermate. Although, due to the history and epidemiological context, we might presume that the cause of death for the later two was the notoedric mange associated with aelurostrongylidosis, no confirmation was possible as no necropsy was performed. Both our patients were in poor body condition and their food consisted mainly of household waste and probably supplemented by game meat. Usually, most of the cats infected with *A. abstrusus* present mild to moderate respiratory signs. However, there are reports of fatal cases in kittens (19).

*Notoedres cati* mites are detected mainly by skin scraping and examination under the light microscope. In these cases, *N. cati* mites were identified in high numbers through skin scraping, coat brushing, acetate tape impression, flotation, and Baermann techniques. The acetate tape impression was found to have similar results with skin scraping, and it was able to isolate the mite from cats with negative skin scrapings (20). Cats can ingest accidentally ectoparasites during grooming and thereafter can be identified in the feces. Thus, a fecal flotation can add value to the diagnosis of ectoparasites when they are not identified to the specific methods as it was already demonstrated for other ectoparasites as *Demodex* spp., *Cheyletiella* spp., *Leporacarus* spp., and *Otodectes cynostic* (21, 22). Also, *N. cati* mites were identified by fecal flotation in asymptomatic stray cats from the USA (22, 23).

Macrocyclic lactones are the treatment of choice for mites, but these molecules can be used also for the treatment of metastrongyloid lungworms (11, 24). Selamectin belongs to this drug group and it is licensed for the treatment of sarcoptic and otodectic manges in dogs and cats. It was also successfully used in the treatment of notoedric mange in different hosts, including cats (25–28). The present cases were treated off-label with selamectin spot-on formulation three times every 2- or 4-week interval. The cats were completely cured for notoedric mange after application of the third treatment. The *A. abstrusus* infection was treated at the same time with notoedric mange in one of the cats, while in the other case, 5 applications of selamectin were needed. The spot-on labeled formulation of fipronil/(S)-methoprene/eprinomectin/praziquantel applied twice monthly before the treatment with selamectin was not effective in treating the notoedric mange or aelurostrongylidosis. According to previous studies, this topical formulation applied once has high efficacy in the treatment of notoedric mange (>99%) and aelurostrongylidosis (90.5%) (29, 30). The failure of the treatment with this product could be attributed to the incorrect application of the product by the owner, the lack of absorption to the skin level, the secondary bacterial infection or the immunosuppression caused by the bad diet, and improper hygienic conditions.

The cats' owners developed pruritic skin lesions after 1 month from the moment when the male cat was housed indoor. It was already demonstrated that *N. cati* has zoonotic potential being diagnosed in humans after close contact with infected cats (31–33). It is a self-limiting, transient infestation characterized by papulovesicular lesions with intense pruritus secondary to hypersensitivity developed to the acarian bite. Generally, the lesions disappear within 2-3 weeks if the contact with the infected cat is interrupted, as the parasite does not multiply on the human skin (32).

In conclusion, notoedric mange and aelurostrongylidosis can be treated successfully with selamectin as a spot-on formulation and the treatment must be continued until no parasite will be detected through specific techniques. However, the success of treatment is conditioned by the improvement of the life quality (nutrition and hygiene) and the treatment of secondary complications.

## Data Availability Statement

The original contributions presented in the study are included in the article/Supplementary Material, further inquiries can be directed to the corresponding authors.

## Ethics Statement

Written informed consent was obtained from the owners for the participation of their animals in this study.

## Author Contributions

AG wrote the manuscript. MOD revised the manuscript and made important suggestions. VM and AU performed the clinical examination of the cats and laboratory methods. AG, GD'A, and MOD collected the parasites and took and prepared the photos for the manuscript. VM designed the manuscript. All authors contributed to the article and approved the submitted version.

## Funding

The work of AG, MOD, AU, and VM was carried out under the frame of the USAMV Cluj-Napoca Internal Grant number 6270/2017. Also, this project was funded by the Ministry of Research and Innovation of Romania, Contract no. PFE/546.

## Conflict of Interest

The authors declare that the research was conducted in the absence of any commercial or financial relationships that could be construed as a potential conflict of interest.

## Publisher's Note

All claims expressed in this article are solely those of the authors and do not necessarily represent those of their affiliated organizations, or those of the publisher, the editors and the reviewers. Any product that may be evaluated in this article, or claim that may be made by its manufacturer, is not guaranteed or endorsed by the publisher.
