# Supplementary material for: Case Report: Notoedric Mange and Aelurostrongylidosis in Two Domestic Cats From Rural Environment in Romania
Source: Front Vet Sci. 2022 Jun 1;9:849525. doi: 10.3389/fvets.2022.849525 (PMC9200890; doi:10.3389/fvets.2022.849525)
Supplement: Supplementary file 1 [file Data_Sheet_1.docx]

## Supplementary files

**Supplementary file 1.** The schedule of the treatments with selamectin (Stronghold®, Zoetis) of the male cat (case 1) and the results of the laboratory examination

|  | **Treatment** | ***N. cati*** | ***A. abstrusus*** |
| --- | --- | --- | --- |
| **Day 0** | 1^st^ | +++ | ++ |
| **Day 14** | 2^nd^ | + | +++ |
| **Day 42** | 3^rd^ | +* | + |
| **Day 63** | - | - | - |

**Legend:** +++ high no. of parasites; ++ moderate no. of parasites; + low no. of parasites; *only one mite was detected to the skin scraping.

**Supplementary file 2.** The schedule of the treatments with selamectin (Stronghold®, Zoetis) of the female cat (case 2) and the results of the laboratory examination

|  | **Treatment** | ***N. cati*** | ***A. abstrusus*** |
| --- | --- | --- | --- |
| **Day -60** | B | CS | CS |
| **Day -30** | B | CS | CS |
| **Day 0** | 1st | +++ | ++ |
| **Day 14** | 2nd | + | +++ |
| **Day 28** | 3th | + | + |
| **Day 56** | 4th | - | + |
| **Day 86** | 5th | - | + |
| **Day 114** | - | - | - |

**Legend:** B – Broadline® (Boehringer Ingelheim) (fipronil, S-methoprene, eprinomectin, and praziquantel); CS – clinical signs; +++ high no. of parasites; ++ moderate no. of parasites; + low no. of parasites.
